# Karyotype of the Blastocoel Fluid Derived by Laser-Assisted Hatching Demonstrates a Low Agreement With the Trophectoderm

**DOI:** 10.3389/fphys.2022.827568

**Published:** 2022-06-08

**Authors:** Liang Wang, Wenjuan Pang, Yi Zhang, Min Hao, Yan Liu, Xiang Wang, Ningxia Sun

**Affiliations:** Center of Reproductive Medicine, Second Affiliated Hospital of Naval Medical University, Shanghai, China

**Keywords:** laser-assisted, blastocoel fluid, trophectoderm, multiple annealing and looping-based amplification cycles, next-generation sequencing

## Abstract

**Objective:**

The aim of this study is to compare the amplification efficiency and the genomic profiles of blastocoel fluid (BF) derived by laser-assisted hatching and trophectoderm (TE) cells derived from the same blastocyst.

**Methods:**

Fifty-four fresh blastocysts underwent shrinkage by laser-assisted hatching, and each BF sample was collected individually. BF and TE cells were retrieved from each blastocyst for chromosome analysis through multiple annealing and looping-based amplification cycles (MALBAC) and next-generation sequencing (NGS).

**Results:**

Fifty-four BF samples and 32 TE samples were retrieved for this study. Out of the 54 BF samples, only 35 provided reliable NGS data for comprehensive chromosome analysis (64.8%), while all 32 TE samples did (100%). Finally, there were 23 pairs of BF and TE samples from the same blastocyst. Only 17.4% of the BF-DNA karyotypes were completely agreeable with the TE samples (4/23).

**Conclusion:**

Blastocoel fluid derived by laser-assisted hatching is easy to operate, and BF-DNA can be successfully amplified and subjected to NGS. Due to the low amplification efficiency and increased discordance with TE, BF does not adequately represent the status of the rest of the blastocyst. The use of BF as a single source of DNA for preimplantation genetic screening (PGS) is not yet advised.

## Introduction

In human-assisted reproductive technology, blastocyst culture has become an effective method for embryo selection. However, even high-quality blastocysts will fail to grow, and one of the principal reasons is chromosome aneuploidy. Therefore, the application of embryo preimplantation genetic screening (PGS) technology can be effective in avoiding repeated implantation failures and recurrent abortions caused by embryo chromosome problems, improving the clinical pregnancy rate, and is widely used in clinical treatment.

Blastocyst trophectoderm (TE) biopsy is a kind of invasive microsurgery. Laser-assisted hatching is generally used in embryo biopsy. Although there are few biopsy cells, it is still damaging to the blastocyst, affecting its later development and reducing the blastocyst implantation rate. Therefore, non-invasive or minimally invasive operations have become a research hotspot in recent years. As early as 2013, some studies found that there was free DNA in the blastocoel fluid (BF), and it was thought that the detection of blastocyst aneuploidy could be completed by detecting the BF (Palini et al., 2013). Gianaroli et al. (2014) studied and compared BF with polar body biopsy and TE biopsy. They found that the consistency of BF and TE biopsy results was as high as 96.6%. Another study provided further evidence that BF is a promising alternative source of DNA for PGT, but the amplification efficiency was about 80% (Gianaroli et al., 2014; Zhang et al., 2016). In addition, the acquisition of BF was also complicated, requiring a high level of micromanipulation. Therefore, simplifying the acquisition of BF and improving its success rate is the key to solving the problem of its application in PGS.

For the acquisition of BF, laser-assisted hatching is the simplest method. To protect the blastocyst from membrane damage arising from ice-crystal formation and to improve its survival after cryopreservation, it is necessary to remove the BF from the blastocyst before vitrification (Mukaida et al., 2006). Therefore, it is a simple and non-invasive method for retrieving BF samples from each blastocyst utilizing laser-assisted hatching, which does not increase any other damage to blastocysts.

In recent years, with the rapid development of whole-genome pre-amplification technology, the efficiency of single-cell amplification has been improved significantly. Xu et al. (2016) successfully detected free DNA in blastocyst culture medium by multiple annealing and looping-based amplification cycles (MALBAC) combined with next-generation sequencing (NGS) technology and applied it to clinical diagnosis, successfully delivering healthy babies. MALBAC has high amplification efficiency, good homogeneity, high coverage, and low release rates for micro DNA or single cells, which is especially suitable for detecting free DNA in BF (Lu et al., 2012).

Therefore, the purpose of this study is to acquire BF by laser-assisted hatching, which is easy to operate with minimal damage to the blastocyst and improves the detection efficiency by using MALBAC combined with NGS technology to explore whether a laser-assisted operation of BF is non-invasive and effective and whether it is suitable for chromosome aneuploidy detection before embryo implantation.

## Data and Methods

### Subjects

Methods: A retrospective analysis of ART cycles was conducted at the Reproductive Medical Center of Shanghai Changzheng Hospital from May 2017 to April 2018. Inclusion criteria: ICSI insemination and at least one blastocyst of 4BB or above was obtained in the fresh cycle. After informed consent of the patients, BF samples of fresh blastocysts were collected. In this study, a total of 54 samples of BF were collected, of which 32 blastocysts underwent trophoblast biopsy for PGS.

### Methods

#### Collection of Blastocoel Fluid

A single fresh blastocyst was transferred to a 20 μL culture media droplet (BM, COOK) (balanced with 6% CO^2^, 5% O^2^, covered by Paraffin oil). The dish was placed on the heating plate of the inverted microscope, the laser energy was adjusted to 300 and shot once at the blastocyst-trophoblast cell junction. The BF was ejected, and the blastocyst began to shrink. The blastocyst was transferred to the incubator (6% CO^2^, 5% O^2^). After 10 min, the collapsed blastocyst was removed with a new Pasteur pipette and vitrified. Then, the remaining droplets containing BF were transferred to a 0.2 ml centrifuge tube (Yikon Genomics Co., Ltd. Shanghai, China), marked with the name, tube number, and date, and then transferred to a −20°C refrigerator for storage after instant centrifugation. It was sent to Yikang Gene Technology Co., for karyotype testing within 48 h.

#### Biopsy of Blastocyst Trophectoderm

For PGS, the collapsed blastocysts were transferred to the original culture drops for other cultures. After 4–6 h, the blastocysts were fully expanded, and some trophoblast cells protruded to form hernias. At this time, the blastocyst was fixed with the holding needle; 6–8 trophoblast cells were aspirated by a biopsy needle and were taken from the blastocyst by laser-assisted hatching. After that, the biopsied cells were thoroughly washed in a G-MOPS solution containing 1% PVP and transferred to a 0.2 ml centrifuge tube (Yikon Genomics Co., Ltd. Shanghai, China), marked with the name, tube number, and date, and then transferred to a −20°C refrigerator for storage after instant centrifugation. It was sent to Yikang Gene Technology Co., for karyotype testing within 48 h. After the biopsy, the blastocysts should be vitrified within 0.5 h.

#### Vitrification of Blastocysts

The Cryotop (Kitazato, Fuji, Japan) was used to vitrify the blastocyst. The patient’s name, medical record number, blastocyst grade, PGS number, and cryopreservation date were marked on the handing part of the Cryotop. The cryosolution VT101 (Kitazato, Fuji, Japan) was returned to room temperature 20–30 min before use. The blastocysts were transferred into the equilibrium solution (ES) and transferred into the VS solution 10–12 min later. The blastocysts were washed three times at the different positions of the droplets and were loaded after 45 s and then put into liquid nitrogen within 60 s.

#### Whole Genome Amplification and Karyotype Analysis of Blastocoel Fluid and Trophectoderm

In this study, ChromInst, One step library method and commercial kits (Yikon Genomics Co., Ltd. Shanghai, China) were used to carry out the whole genome amplification (WGA) and karyotype analysis of BF and TE. The BF or blastocyst TE were transferred into the sample preservation solution (XK-005). Amplify and build a library immediately according to the operation method of the kit. The final library concentration was >1 ng/μ L, and the main band was distributed between 200 and 1,000 bp. After the samples are pooled, the library is sequenced on Illumina nextseq 550 in the way of SE55 to ensure that each sample can obtain a data volume of 1.5 m reads. Chromogo v2.0 bioinformatics analysis was performed on each sample. After a series of data cleaning, bin statistics and GC correction, the blastocyst cavity fluid and chromosome aneuploidy results of TE were finally obtained.

### Statistical Analysis

Statistical calculations were performed using SPSS 19.0 statistical software. BF and TE amplification success rates were assessed using a chi-square test. The difference in the number of affected chromosomes between the embryo biopsies was considered to be statistically significant when the *P*-value was < 0.05.

## Results

### Whole Genome Amplification of Blastocoel Fluid - and Trophectoderm-DNA for Next-Generation Sequencing Analysis

**FIGURE 1:**
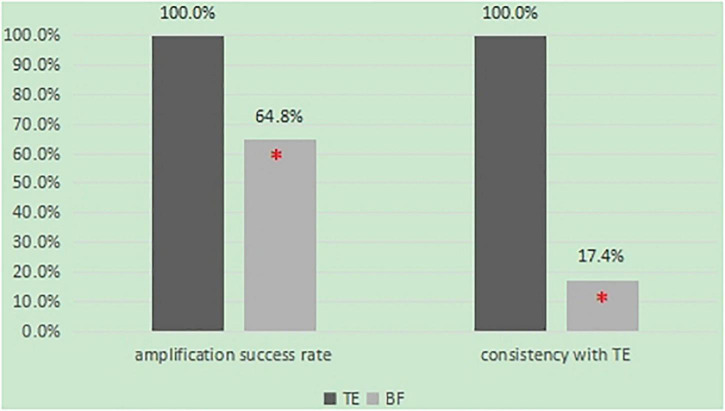
The comparison of BF and TE in the amplication success rate and consistency.

From May 2017 to April 2018, a total of 54 BF samples from fresh blastocysts and 32 TE samples were collected and submitted for WGA. After WGA, a sufficient amount of DNA was detected in all TE samples (100%) but in only 36 of the 54 BF biopsies (64.8%). There was a difference in the amplification success rate between BF and TE (64.8 vs. 100%, *P* < 0.05) (Figure 1).

### Comparison of Karyotype Consistency Between Blastocoel Fluid and Trophectoderm Samples

In this study, 54 BF samples and 32 TE samples were collected. Only 23 pairs of BF samples and TE biopsy retrieved from the same blastocyst could be matched. As shown in Table 1, the false positive rate of BF detection was as high as 75%, the detection accuracy rate was only 47.8%, and the specificity was 25.0%. Among them, four cases were completely consistent with the results of the TE biopsy (Table 2). The consistency was as low as 17.4% (4/23) (Figure 1), and the sex chromosomes of BF in the two cases were not consistent with the results of the TE biopsy. However, the results of this study showed that the detection results of BF-DNA were inconsistent with TE, and most showed multiple chromosome aneuploidies. As shown in Table 3, compared with the karyotype of TE, there were 16 cases of multiple chromosome aneuploidies in 23 samples (69.6%) (Table 3), which indicated that there might be contamination in the BF-DNA.

**TABLE 1:** Whole-genome amplification of blastocoel fluid (BF)- and trophectoderm (TE)-DNA for next-generation sequencing (NGS) analysis.

TE				
	**Abnormal (Positive)**		**Normal (Negative)**	
	
BF	True positive A	7	False positive B	12
	False negative C	0	True negative D	4
	A + C	7	B + D	16

Positive predictive value = 100% × [A/(A + B)]				36.8%
Negative predictive value = 100% × [D/(C + D)]				100.0%
False positive = B/(B + D)				75.0%
False negative = C/(A + C)				0.0%
Detection accuracy = 100% × [(A + D)/N]				47.8%
Sensitivity = A/(A + C)				100.0%
Specificity = D/(B + D)				25.0%

**TABLE 2:** Molecular karyotypes of blastocoel fluid (BF) and trophectoderm (TE) being consistent.

	TE	BF
LM_1	46,XN	46,XN
XLJ_2	46,XX	46, XY,−Y(× 0,mos*)
ZJY_1	46,XN	46,XN
ZJY_2	46,XX	46, XY, + X(× 2,mos*),−Y(× 0,mos*)

*Suspected mosaic.

**TABLE 3:** Molecular karyotypes of blastocoel fluid (BF) and trophectoderm (TE).

	TE	BF
E1	46,XN	Multiple chromosome aneuploidies, −4(× 1), + 5(pter→q23.2,∼121 M, × 3,mos*), + 6(p23→qter,∼149 M, × 3,mos*), + 7(p14.3→qter,∼123 M, × 3,mos*),−12(p12.1→qter,∼107 M, × 1),…
E2	46,XN	Multiple chromosome aneuploidies, −1(pter→q25.2,∼170 M, × 1,mos*), + 2(p24.1→q14.1,∼98 M, × 3,mos*),−3p(pter→p12.3,∼80 M, × 1,mos*),−3(p12.1→qter,∼113 M, × 1,mos*), + 4(× 3,mos*),…
E3	45,XN,−16(× 1)	Multiple chromosome aneuploidies, + 4(× 3,mos*), + 5(× 3,mos*),−6(p21.2→qter,∼134 M, × 1,mos*), + 7(× 3,mos*),−16(× 1,mos*),…
E4	46,XN	46,XN,−X(pter→→q12,∼66 M, × 1,mos*),−4(pter→q22.1,∼91 M, × 1,mos*),−4q(q22.3→qter,∼95 M, × 1,mos*), + 10(pter→q21.2,∼62 M, × 3,mos*)
LM_3_C	46,XN	Multiple chromosome aneuploidies, + 1(× 3),−5(× 1,mos*), + 8(× 3,mos*),−13(× 1,mos*), + 15(× 3),…
XLJ_1_C	46,XN	Multiple chromosome aneuploidies, −5(× 1,mos*), + 15(× 3,mos*), + 17(× 3,mos*), + 18(pter→q22.1,∼64 M, × 3,mos*),−19(× 1,mos*),.
XLJ_3_C	46,XN	Multiple chromosome aneuploidies, + 1(× 3,mos*),−2(× 1), + 8(pter→q21.12,∼79 M, × 3,mos*), + 11(× 3), + 15(× 3),…
ZJY_2_C	46,XN	46, XY, + X(× 2,mos*),−Y(× 0,mos*)
WMY_1_C	46,XN	Multiple chromosome aneuploidies, −2(× 1,mos*),−11(× 1,mos*), + 14(× 3,mos*),−15(× 1,mos*),−18(× 1),…
WMY_2_C	47,XN, + 16(× 3)	46,XY, + X(× 2,mos*),−Y(× 0,mos*), + 16(× 3,mos*)
CXF_1_C	46,XN, + 22(× 3,mos*,∼70%)	Multiple chromosome aneuploidies, −1q(q23.3→qter,∼82 M, × 1,mos*), + 4(pter→q13.1,∼67 M, × 3,mos*), + 5q(q13.2→q33.3,∼89 M, × 3,mos*), + 17(× 3), + 22(× 3)…
CXF_2_C	47,XN,−7q(q35→q36.3,∼12 M, × 1), + 16(× 3)	Multiple chromosome aneuploidies, −7(q35→qter,∼15 M, × 1), + 11(× 3,mos*),−15(× 1,mos*), + 17(× 3,mos*), + 18(× 3,mos*)
CXF_3_C	46,XN	46,XN, + 20(× 3,mos*,∼70%)
XJ_1_C	46,XN	Multiple chromosome aneuploidies, −1p(p33→p13.1,∼71 M, × 1,mos*), + 6q(q15→qter,∼83 M, × 3),−9(p21.1→qter,∼104 M, × 1,mos*), + 10q(q21.3→qter,∼70 M, × 3,mos*), + 11(pter→q22.1,∼101 M, × 3,mos*),…
WYX_1_C	46,XN	Multiple chromosome aneuploidies, −1(× 1,mos*), + 3(× 3),−6(× 1,mos*),−7(× 1,mos*), + 9(× 3)
WYX_2_C	46,XN	46,XY, + X(× 2,mos*),−Y(× 0,mos*), + 6(pter→q16.1,∼97 M, × 3,mos*)

*Suspected mosaic.

## Discussion

Many studies have shown that the proportion of aneuploidy embryos is higher in women older than 35 years, with recurrent abortions and implantation failures and severe male infertility (Lathi et al., 2008; Kushnir and Frattarelli, 2009; Rubio et al., 2013). It is not very effective to select the transferred embryos only from the morphology and developmental dynamics. The application of PGS can significantly improve the clinical pregnancy rate of these infertile patients. It is generally believed that blastocyst TE biopsy is an effective method for PGS. Occasionally, cell apoptosis, loss, or other errors will lead to amplification failure, and the probability is very low (Tobler et al., 2015). However, blastocyst TE biopsy is an invasive operation. Commonly, 4∼8 cells were derived from blastocysts by laser-assisted hatching. More cells will be lost or damaged by the invasive procedure, which inevitably affects the late development of blastocysts and reduces the blastocyst seeding rate.

Therefore, the application of BF as a non-invasive or minimally invasive method has gradually concerned researchers. Since 2013, when it was first reported that there was a trace of DNA in BF, researchers have done many studies on testing the DNA content in BF, the optimization of the DNA amplification method, and the accuracy and consistency of amplification results. However, there are still many problems to be solved about BF used in chromosome aneuploidy detection before embryo implantation.

First of all, it is difficult to separate and collect BF with DNA content in it. Most studies reported that the collection of BF was completed with the micromanipulation system, and the BF was extracted by injecting a needle into the blastocyst cavity (D’Alessandro et al., 2012; Magli et al., 2016). However, if we want to collect enough BF with more DNA, we need to wait until the blastocyst cavity completely shrinks. At this time, the injection needle can easily damage the inner cell mass, which requires a high level of operational skills and great operation cost. This study is the first to use laser-assisted collapsing of the blastocyst to collect BF. The operation is simple, and the blastocysts need to be shrunk before cryopreservation, which does not increase the damage to the blastocysts. Therefore, BF derived by laser-assisted hatching is thought to be a non-invasive procedure.

Secondly, the content of DNA in BF and the rate of amplification were low. The efficiency of WGA in BF reported in the other studies is 63∼82%, which is lower than the success rate of blastocyst TE amplification (Wang et al., 2012; Gianaroli et al., 2014; Tobler et al., 2015; Magli et al., 2016). In this study, MALBAC was used, which has a high sensitivity. A single cell, single chromosome, or 0.5 pg genomic DNA can be amplified (Lu et al., 2012; Wang et al., 2012). However, our results showed that the amplification rate of BF was low (64.8%), significantly lower than that of blastocyst TE (100%), which greatly limited the application of BF in embryo chromosome aneuploidy screening.

Finally, the DNA karyotype of the blastocysts BF was much different from that of blastocysts. Early studies have shown that the consistency of DNA karyotype in BF with TE biopsy or the whole blastocyst is low, only 25–33% (Perloe et al., 2013; Poli et al., 2013). Some reports showed that BF- and TE-DNA karyotype consistency could be as high as 97% (Gianaroli et al., 2014; Magli et al., 2016). However, other studies found that there is no good consistency in the DNA karyotype of BF and TE. Tobler et al. (2015) showed that the consistency of the DNA karyotype of BF and the blastocyst was 62%. Tšuiko et al. (2018) recently found that the consistency of DNA karyotype of BF with that of TE and inner cell mass was lower than 40%. In our study, the consistency of DNA karyotype between BF and TE was as low as 17.4%. There were some reasons for this. First, the source of DNA in the BF is contaminated, which may come from fragments, polar bodies, apoptotic cells, dead cells, and cell-free DNA from trophoblast cells and inner cell mass (Hammond et al., 2016). Furthermore, a WGA study showed that 63.6% of blastocysts expelled cell debris with abnormal chromosomal rearrangements. According to the statistics, 55.5% of euploid blastocysts expel aneuploid debris, strongly suggesting that the primary source of cell-free DNA in culture media is expelled aneuploid blastomeres and/or their fragments. It is also in BF, which leads to high rates of false-positive diagnoses of human embryos, resulting in embryos with an entirely normal pregnancy potential being labeled for non-use and/or disposal (Orvieto et al., 2021). Our study also showed higher rates of false-positive diagnoses in BF, as shown in Table 3. Studies have shown that the false positive rate of whole gene amplification by SurePlex method is lower than that by MALBAC (Deleye et al., 2015). It is speculated from our results that reducing the false positive rate will improve the consistency of BF and TE results. Finally, laser energy can directly damage the DNA structure and lead to DNA strand breakage, resulting in a significant increase in the proportion of aneuploid debris in BF, as shown in Table 3.

In conclusion, BF derived by laser-assisted hatching is easily operated, and BF-DNA can be successfully amplified and subjected to NGS. Due to the low amplification efficiency and increased discordance with TE, BF does not adequately represent the status of the rest of the blastocyst. The use of BF as a single source of DNA for PGS is not yet advised. The clinical application of BF needs to be further studied and demonstrated.

## Data Availability Statement

The original contributions presented in the study can be accessed through Figshare (doi: 10.6084/m9.figshare.19840036), further inquiries can be directed to the corresponding author/s.

## Ethics Statement

The studies involving human participants were reviewed and approved by the Ethics Committee of Second Affiliated Hospital of Naval Medical University. The patients/participants provided their written informed consent to participate in this study.

## Author Contributions

LW, WP, and YZ: conception and design of the research, and writing of the manuscript. MH and YL: acquisition of data. XW: analysis and interpretation of the data. NS: statistical analysis. LW: obtaining financing. LW and NS: critical revision of the manuscript for intellectual content. All authors read and approved the final draft.

## Conflict of Interest

The authors declare that the research was conducted in the absence of any commercial or financial relationships that could be construed as a potential conflict of interest.

## Publisher’s Note

All claims expressed in this article are solely those of the authors and do not necessarily represent those of their affiliated organizations, or those of the publisher, the editors and the reviewers. Any product that may be evaluated in this article, or claim that may be made by its manufacturer, is not guaranteed or endorsed by the publisher.
